# Prospective monitoring of *in vitro* produced PR3-ANCA does not improve relapse prediction in granulomatosis with polyangiitis

**DOI:** 10.1371/journal.pone.0182549

**Published:** 2017-08-03

**Authors:** Judith Land, Wayel H. Abdulahad, Suzanne Arends, Jan-Stephan F. Sanders, Coen A. Stegeman, Peter Heeringa, Abraham Rutgers

**Affiliations:** 1 Department of Rheumatology and Clinical Immunology, University of Groningen, University Medical Center Groningen, Groningen, the Netherlands; 2 Department of Internal Medicine, Division of Nephrology, University of Groningen, University Medical Center Groningen, Groningen, the Netherlands; 3 Department of Pathology and Medical Biology, University of Groningen, University Medical Center Groningen, Groningen, the Netherlands; Keio University, JAPAN

## Abstract

**Objectives:**

Patients with granulomatosis with polyangiitis (GPA) are prone to disease relapse. Currently, no good biomarkers are available to predict relapses in individual patients. This study aimed to determine whether patients at risk for relapse can be distinguished based on increased *in vitro* autoantibody production.

**Methods:**

Eighty-four proteinase 3 (PR3) anti-neutrophil cytoplasmic antibody (ANCA) positive GPA outpatients were prospectively monitored for up to two years and 32 healthy controls were included. At periodic intervals peripheral blood mononuclear cells were isolated, cultured and *in vitro* production of total and PR3-ANCA-specific IgG was determined. Moreover, serum ANCA titers were measured by indirect immunofluorescence.

**Results:**

Sixteen patients (21%) relapsed during the follow-up period. At time of inclusion no significant differences were present for ANCA production between relapsing and non-relapsing patients. Samples before relapse exhibited increased serum ANCA titers and *in vitro* PR3-ANCA IgG levels compared with inclusion samples from non-relapsing patients. When evaluating changes over time, increasing serum ANCA titers were observed prior to relapse compared to a 1-year follow-up from non-relapsing patients. No significant change in *in vitro* PR3-ANCA levels occurred prior to relapse, compared to non-relapse patients.

**Conclusions:**

While differences were observed for the serum ANCA titer in relapsing and non-relapsing patients, monitoring *in vitro* PR3-ANCA IgG production does not improve relapse prediction in GPA patients.

## Introduction

Granulomatosis with polyangiitis (GPA) is one of the anti-neutrophil cytoplasmic antibody (ANCA) associated vasculitides (AAV), forms of vasculitis that predominantly affect small blood vessels in the respiratory tract and kidneys [1]. In GPA patients, ANCA are mainly directed against proteinase 3 (PR3). Clinical and experimental evidence demonstrates a crucial role for the autoantibodies in disease pathogenesis [1,2]. Patients with AAV are prone to disease relapse, resulting in progressive loss of organ function and increased burden of co-morbidities [3]. Maintenance therapy aimed at preventing (early) disease relapse comes at the cost of treatment related morbidity and expense [4,5]. There is a clear need for biomarkers that can distinguish patients susceptible for disease relapse. Patient groups at increased risk for relapse include those that have lung involvement [6], and patient that present with chronic nasal carriage of *Staphylococcus aureus* [7]. Nevertheless, an accurate method to predict relapses in individual patients is currently not available.

One potential biomarker that has been thoroughly investigated is monitoring of serum ANCA titers. However, results from numerous studies are inconsistent and monitoring ANCA titers is only modestly predictive for relapse [8–10]. Previously, we have demonstrated that it is possible to induce PR3-ANCA production using an *in vitro* system [11,12] based on stimulation of peripheral blood mononuclear cells (PBMCs) and postulated that this may be a more accurate reflection of the ongoing pathogenic process and active ANCA production in GPA patients.

In the current study we aimed to determine whether GPA patients at risk for relapse can be distinguished based on increased (*in vitro)* autoantibody production. To investigate this, we performed a prospective cohort study in 84 PR3-ANCA positive GPA patients in the setting of daily clinical practice. In this cohort we monitored *in vitro* PR3-ANCA IgG production, as well as the serum ANCA titer and compared their value for predicting an ensuing disease relapse.

## Materials and methods

### Study population

Between 2013 and 2015 84 consecutive GPA outpatients from the University Medical Center Groningen (UMCG) and 32 healthy controls (HC) were included. The diagnosis of GPA was based on definitions outlined in the Chapel Hill Consensus Conference and patients fulfilled the classification criteria of the American College of Rheumatology [13,14]. Patients were included irrespective of disease status. At time of inclusion 81 patients were in clinical remission, with remission being defined as an absence of any clinical and/or laboratory signs that could be attributed to active disease. Three patients had active disease with a BVAS > 0 at inclusion, but achieved remission prior to their second visit. Of these three patients, one relapsed during follow up, this second relapse was preceded by at least eight months of full remission. All patients were confirmed positive for PR3-ANCA at least once during their disease course. GPA patients were monitored for up to two years of follow-up and samples were collected according to the normal visit schedules in daily clinical practice. HC were included at a single time point. Characteristics of the patient and control populations at time of inclusion are listed in Table 1. Relapses were based on clinical and laboratory evaluation and had to result in the decision to initiate or increase immunosuppressive therapy. All patients had a positive BVAS (range 2–19) at time of relapse. Clinical data of the individual patients at time of relapse is listed in S1 Table. Median time between last sampling and relapse was 0.15 (range 0.04–0.53) years. All subjects gave informed written consent and the study was approved by the Medical Ethics Committee of the UMCG.

**Table 1:** Patient and healthy control characteristics.

	HC	GPA	GPA–no relapse during follow-up	GPA–relapsed during follow-up
subjects, n (% male)	32 (53)	84 (43)	68 (43)	16 (44)
Age, mean (range)	56.8 (44–74)	58.6 (26–83)	60.2 (26–84)	55.0 (32–76)
PR3-ANCA titer, median (range)		1:80 (0–1:640)	1:40 (0->640)	1:80 (0->640)
Creatinine umol/L, median (range)		87 (57–409)	87 (57–409)	93 (61–171)
eGFR ml/min*1,73m^2^, median (range)		68 (13–111)	65.5 (13–111)	71 (26–95)
CRP mg/L, median (range)		4 (0.3–72)	4 (0.3–72)	4 (0.4–20)
Disease duration in years, median (range)		9.5 (0.2–42)	8.8 (0.2–42)	14.9 (2–24)
Number of total relapses, median (range)		1 (0–10)	1 (0–6)	4 (1–10)
BVAS, median (range)		0 (0–10)	0 (0–10)	0 (0–2)
Clinical manifestations, n (%)				
Renal		51 (61)	40 (59)	11 (69)
ENT		61 (73)	49 (72)	12 (75)
Joints		44 (52)	34 (50)	10 (63)
Pulmonary		56 (67)	41 (60)	15 (94)
Nervous system		26 (31)	22 (32)	4 (25)
Eyes		30 (36)	22 (32)	8 (50)
Cutaneous		18 (21)	14 (21)	4 (25)
Other		8 (10)	5 (7)	3 (19)
Disease form, n (%)				
Localised		3 (4)	3 (4)	0 (0)
Early systemic		13 (16)	11 (16)	2 (13)
Generalised		55 (66)	43 (63)	12 (75)
Severe		13 (16)	11 (16)	2 (13)
Treatment at time of sampling, n (%)				
Aza		9 (11)	5 (7)	4 (25)
Pred		9 (11)	8 (12)	1 (6)
Aza + pred		16 (19)	13 (19)	3 (19)
MMF + pred		8 (10)	4 (6)	4 (25)
MTX		1 (1)	1 (2)	0 (0)
No immunosuppressive therapy		41 (49)	37 (54)	4 (25)
Induction therapy, n (%)				
Cyc, pred		57 (68)	47 (69)	10 (63)
Cyc, pred, followed by RTX		8 (10)	4 (6)	4 (25)
Cyc, pred, plasmapheresis		13 (15)	11 (16)	2 (13)
Pred, MTX		2 (2)	2 (3)	0 (0)
Co-trimoxazole		4 (5)	4 (6)	0 (0)

ANCA, anti-neutrophil cytoplasmic antibody; Aza, azathioprine; BVAS, Birmingham Vasculitis Activity Score; CRP, C-reactive protein; Cyc, cyclophosphamide; eGFR, estimated glomerular filtration rate; GPA, granulomatosis with polyangiitis; HC, healthy control; MMF, mycophenolate mofetil; MTX, methotrexate; pred, prednisolone; RTX, rituximab

### Quantification of *in vitro* produced total and PR3-ANCA specific IgG

PBMC isolation, culture, and quantification of total IgG and PR3-ANCA IgG levels was performed as described previously [12]. Briefly, lithium-heparinized venous blood was obtained from patients and HC. PBMC were isolated using Lymphoprep (Axis-Shield, Oslo, Norway). Cells were resuspended at a concentration of 10^6^ cells/mL in Roswell Park Memorial Institute (RPMI) medium (Lonza, Basel, Switzerland), supplemented with 50 μg/mL gentamicin (GIBCO, Life Technologies, Grand Island, NY, USA) and 10% fetal calf serum (FCS, Lonza) and cultured with or without 3.2 μg/mL CpG-ODN 2006 (Hycult Biotech, Uden, the Netherlands), 100 ng/mL BAFF (PeproTech Inc., Rocky Hill, NJ, USA) and 100 ng/mL IL-21 (Immunotools, Friesoythe, Germany) at 37°C with 5% CO2. After 12 days, the culture supernatants were collected and stored at -20°C. Levels of PR3-ANCA IgG in the culture supernatants were determined using Phadia ImmunoCAP® 250 analyser with EliA PR3S (Thermo Fisher Scientific, Waltham, MA, USA) and are expressed in response units (RU). Levels of total IgG were measured using an in-house ELISA and are expressed in ng/mL. For PR3-ANCA IgG, levels measured in HC were used to calculate a positive cut-off value, based on mean + 3 times the standard deviation in order to determine production of significant levels of *in vitro* PR3-ANCA IgG in GPA patients.

### Serum ANCA titer

ANCA detection was performed by indirect immunofluorescence, as described previously [15]. Serum samples were tested at 2-fold serial dilutions starting at 1:20.

### Statistical analysis

Statistical analysis was performed using SPSS v22 (IBM Corporation, Chicago, IL, USA) and Graphpad Prism v5.0 (GraphPad Software, San Diego, CA, USA). ANCA production was compared between 1) inclusion samples of GPA patients and healthy controls, 2) inclusion samples of relapsing and non-relapsing patients and 3) samples before relapse and inclusion samples of non-relapsing patients using the Mann-Whitney U test.

Further analyses were performed to evaluate changes over time in relapsing versus non-relapsing patients. For relapsing patients, the mean difference between inclusion and the last sample taken before relapse was 0.8 ± 0.5 years. For non-relapsing patients this time frame was matched as accurately as possible. Since the regular sample frequency was every 6 months, samples closest to 1-year follow-up were used (mean 1.0 ± 0.2 years). The change in ANCA production over time (from inclusion to relapse or 1-year follow-up; Δ0–1) was compared between relapsing and non-relapsing patients using the Mann-Whitney U test. One patient relapsed within one month of inclusion and was excluded fully from this analysis. For another two relapsing and four non-relapsing patients, samples were only available at inclusion and they were excluded from the change in ANCA analysis. P-values <0.05 were considered statistically significant.

## Results

### *In vitro* production of total and PR3-ANCA specific IgG

**Fig 1:**
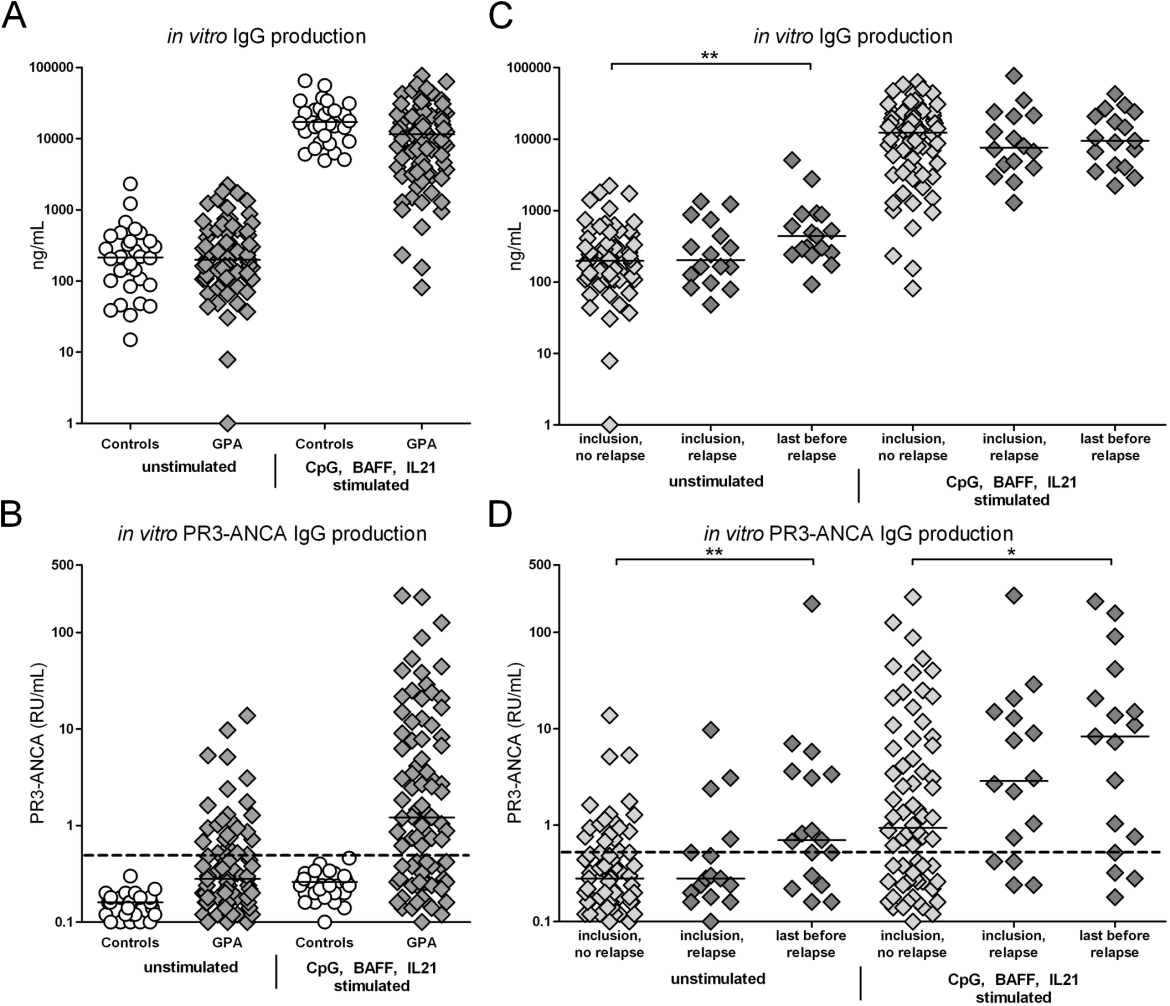
*In vitro* total and PR3-ANCA specific IgG production. Levels of **A)** total IgG and **B)** PR3-ANCA specific IgG production were determined in *in vitro* culture supernatant samples from 84 GPA patients and 32 healthy controls at time of inclusion. **C-D)** The GPA patients were divided based on future relapse. Results at time of inclusion from patients with and without relapse during follow-up and the last sample taken before relapse are depicted. The PR3-ANCA IgG levels in healthy controls were very low and considered to be negative. The dashed line represents the positive cut-off, based on the mean of the healthy control samples + 3 times the standard deviation. Horizontal lines represent median values. *p<0.05. **p<0.01.

Total and PR3-ANCA specific IgG was measured in cell culture supernatants from 84 patients and 32 healthy controls. At time of inclusion, levels of IgG were similar in patients and controls for both unstimulated and CpG, BAFF and IL21 stimulated samples (Fig 1A). In 30% of the patients, significant levels of PR3-ANCA IgG were observed in unstimulated samples and 64% of patients were found positive for *in vitro* ANCA production in CpG, BAFF and IL21 stimulated samples (Fig 1B). When comparing patients with or without immunosuppressive treatment at time of sample collection, total *in vitro* produced IgG levels were significantly decreased in patients on maintenance therapy while PR3-ANCA specific IgG levels were not significantly affected by current treatment (S1 Fig).

During the prospective study 16 GPA patients (21%) relapsed. At time of inclusion no significant differences were observed for *in vitro* total or PR3-ANCA specific IgG production in patients with or without a future relapse. However, when comparing the last sample taken before relapse with inclusion samples of non-relapsing patients, a significantly higher production of both total and PR3-ANCA specific IgG was observed in unstimulated samples from relapsing patients. Furthermore, in samples stimulated with CpG, BAFF and IL21 higher levels of PR3-ANCA IgG were present in relapsing patients compared to patients that did not relapse during the study (Fig 1C and 1D). As has been previously described, serum ANCA titers did not differ at time of inclusion (*p = 0*.*27*) and were increased prior to relapse (*p = 0*.*016*) compared to inclusion samples of non-relapsing patients (S2 Fig).

The biomarkers did demonstrate a high level of variability before relapse. Serum ANCA titers appeared to increase in most patients before relapse. With respect to *in vitro* PR3-ANCA production, a number of patients clearly had an increasing production before they relapsed while others remained relatively stable or were consistently negative. This was seen in both unstimulated and CpG, BAFF and IL21 stimulated samples. In samples from non-relapsing patients, the *in vitro* PR3-ANCA levels did not remain stable over time either, but exhibited general variation between sampling time points (S3 Fig).

### Changes in ANCA production over time in relapsing versus non-relapsing GPA patients

The difference between the inclusion samples and last sample taken before relapse was compared to the difference between inclusion and the 1-year sample in patients without a relapse during the study. Three patients relapsed immediately after the inclusion sample and could not be included in this analysis. The remaining 13 patients relapsed after a mean follow-up of 0.8 years. As mentioned before, no significant differences were observed for *in vitro* ANCA production at time of inclusion. The change in serum ANCA titer between inclusion and relapse was significantly different compared to the change during 1 year of follow-up in patients without relapse. Specifically, an increase in serum ANCA titer occurred prior to relapse while this change was not present in patients without a relapse. Moreover, when samples taken before relapse were compared to 1-year samples in non-relapsing patients, significantly higher serum ANCA titers were found in relapsing patients. There was also a higher production of *in vitro* ANCA in unstimulated culture samples before relapse, although due to large variation the change over time was not significantly different from non-relapsing patients (Table 2).

**Table 2:** Changes in ANCA production over time.

*in vitro* ANCA unstim			n	mean	SD	median	IQR	p-value
	t = 0	no relapse	68	0.76	1.84	0.28	0.16–0.65	0.84
		relapse	15	1.07	2.47	0.28	0.18–0.52	
	t = 1	no relapse	64	1.30	5.60	0.35	0.20–0.65	**0.05**
		relapse	13	16.84	54.47	0.70	0.26–4.71	
	Δ0–1	no relapse	64	0.51	5.10	0.06	-0.06–0.20	0.10
		relapse	13	15.66	54.11	0.34	-0.02–1.97	
***in vitro* ANCA stim**								
	t = 0	no relapse	68	12.26	34.22	0.94	0.26–6.61	0.19
		relapse	15	22.11	61.23	2.68	0.42–12.88	
	t = 1	no relapse	64	16.17	35.98	1.59	0.43–12.28	0.43
		relapse	13	38.78	69.63	7.32	0.42–52.22	
	Δ0–1	no relapse	64	3.40	21.35	0.20	-0.37–5.28	0.51
		relapse	13	14.94	59.72	2.18	-0.17–5.37	
**ANCA titer**								
	t = 0	no relapse	68	109.7	168.6	40	0–160	0.20
		relapse	15	146.7	204.9	80	40–160	
	t = 1	no relapse	64	88.4	136.8	40	0–80	**0.003**
		relapse	13	192.3	178.8	80	80–320	
	Δ0–1	no relapse	64	-14.4	84.1	0	-20–0	**0.04**
		relapse	13	32.3	147.8	20	0–100	

ANCA, anti-neutrophil cytoplasmic antibody; IQR, interquartile range; PR3, proteinase 3; SD, standard deviation; stim, cell culture samples stimulated with CpG-oligodeoxynucleotides, B cell activating factor and interleukin-21; unstim, unstimulated cell culture samples.

## Discussion

One of the main challenges in the clinical care of GPA patients is to determine which patients are at risk for relapse, as no clear individual predictive factor has been found so far. Here, we investigated the potential value of monitoring (*in vitro)* PR3-ANCA production by peripheral blood mononuclear cells in predicting an ensuing relapse in GPA patients.

For a number of patients, a clear increase of *in vitro* ANCA production was observed prior to relapse. However, there were also relapsing patients that remained negative for *in vitro* PR3-ANCA throughout the follow-up period, or were positive but relatively stable over time. On group level, the *in vitro* ANCA production in relapsing patients was not significantly different from non-relapsing patients, nor was the change before relapse. One problem with using *in vitro* ANCA production to predict future relapses appears to be the large variation in patient responses. This indicates that *in vitro* ANCA production is not a usable marker for relapse prediction in individual GPA patients in daily clinical practice.

This marker could still be of potential interest if the right subpopulation of patients could be identified. Our analysis did not have sufficient power to make specific sub-analyses, for example for renal involvement patients only, where the ANCA titer was proven more accurate for predicting relapse [16]. Moreover, it was suggested recently that the association between PR3-ANCA and relapse is affected by the detection method used to determine ANCA levels, as well as the patient-specific clinical context. It was demonstrated that serial PR3-ANCA testing may be useful for prediction of severe relapses in patients with renal involvement or alveolar haemorrhage, or in patients treated with rituximab [17].

In contrast to *in vitro* ANCA production, the serum ANCA titer was increased in the majority of patients before relapse, and demonstrated an increase over time not seen in non-relapsing patients. Serum ANCA titers are easier to determine than *in vitro* ANCA production and are often already routinely measured by indirect immunofluorescence (IIF) [18]. Nonetheless, there were several patients in our study that did not demonstrate an increase in serum ANCA titer prior to relapsing, and it has been suggested that other methods than IIF may be more sensitive for ANCA detection [19]. One example could be measuring sequential serum samples with the Phadia ImmunoCAP system that was used for culture supernatants in our study, which is currently under investigation and may provide more reliable results than IIF and improve the predictive value of serum ANCA titers.

There are a few limitations to our study. The number of relapses detected during follow-up was low for drawing definitive conclusions. The variation in time between sampling moments in individual patients and the time between the last collected sample and actual relapse is another point that makes data analysis challenging. In order to reduce potential bias related to variation in follow-up time at group level, we matched this time frame for non-relapsing patients to the time before relapse of the relapsing group. Finally, while we were mainly interested in changes before relapse, it would be interesting to see how these data compare to the actual moment of relapse.

In summary, we report that the majority of GPA patients in our study demonstrated an increasing serum ANCA titer prior to relapse. While a select number of patients had a clear increase in *in vitro* PR3-ANCA production prior to relapse, this method cannot be applied to improve relapse prediction in individual GPA patients in daily clinical practice.

## Supporting information

S1 Table GPA patient characteristics at time of relapse. ANCA, anti-neutrophil cytoplasmic antibody; Aza, azathioprine; BVAS, Birmingham Vasculitis Activity Score; CRP, C-reactive protein; Cyc, cyclophosphamide; ENT, ear, nose and throat; IIF, indirect immunofluorescence; MMF, mycophenolate mofetil; pred, prednisolone; RTX, rituximab.
(DOCX)

S1 Fig Effect of current treatment on IgG production. Graphs represent data of 84 GPA patients. Patients were divided based on whether they received immunosuppressive treatment. Patients classified as untreated are those that received no immunosuppression at time of sampling, all patients had received treatment in the past. All types of immunosuppressive treatment were combined in the treated group. **A)** Total IgG production was decreased in patients currently receiving treatment, while **B)** PR3-ANCA production was not significantly affected by current treatment. Horizontal lines represent median values. *p<0.05, **p<0.001.
(TIF)

S2 Fig ANCA titer in relapsing and non-relapsing patients. Graphs represent data of 84 GPA patients. Patients are divided based on whether they relapsed during the study period. In the left panel relapse and non-relapse patients are compared at time on inclusion, in the right panel after about 12 months.
(TIF)

S3 Fig Changes in ANCA production in relapsing and non-relapsing GPA patients. Results of all measured time points for A) ANCA titer, B) *in vitro* ANCA production in unstimulated culture samples and c) *in vitro* ANCA production in culture samples stimulated using CpG, BAFF and IL21 for individual patients. Graphs on the left represent 16 relapsing patients. Graphs on the right represent all non-relapsing patients with at least 3 samples during follow-up (n = 51).
(TIF)

S1 File Data file. Measurement and clinical data for all analysed timepoints in GPA patients and HC.
(XLSX)
