# Supplementary material for: Prospective monitoring of in vitro produced PR3-ANCA does not improve relapse prediction in granulomatosis with polyangiitis
Source: PLoS One. 2017 Aug 3;12(8):e0182549. doi: 10.1371/journal.pone.0182549 (PMC5542648; doi:10.1371/journal.pone.0182549)
Supplement: S1 Table — ANCA, anti-neutrophil cytoplasmic antibody; Aza, azathioprine; BVAS, Birmingham Vasculitis Activity Score; CRP, C-reactive protein; Cyc, cyclophosphamide; ENT, ear, nose and throat; IIF, indirect immunofluorescence; MMF, mycophenolate mofetil; pred, prednisolone; RTX, rituximab. (DOCX) [file pone.0182549.s001.docx]

**S1 Table. GPA patient characteristics at time of relapse.**

| **Patient** | **BVAS** | **Organ involvement** | **CRP** (**mg/L)** | **Creatinine** (**umol/L)** | **ANCA titer (IIF)** | **Treatment** |
| --- | --- | --- | --- | --- | --- | --- |
| 1 | 13 | renal/joints | 110 | 270 | 320 | CYC/pred |
| 2 | 10 | ENT/lung | 10 | 68 | 80 | MMF/pred |
| 3 | 8 | renal/joints | 43 | 102 | >640 | RTX/pred |
| 4 | 15 | renal/joints | 9,3 | 186 | >640 | CYC/pred |
| 5 | 9 | cardiac/joints | 147 | 66 | 160 | Pred/aza |
| 6 | 18 | ENT/eye/renal | 4,3 | 139 | 80 | Pred/RTX |
| 7 | 5 | lung | 51 | 94 | 160 | Pred/RTX/MMF |
| 8 | 8 | lung/ENT | 6,3 | 72 | 80 | Pred/MMF |
| 9 | 13 | ENT/joints/renal | 9,6 | 151 | 80 | Pred/RTX |
| 10 | 9 | skin/renal | 18 | 67 | >640 | Pred/CYC |
| 11 | 4 | ENT | 2,1 | 101 | 160 | Pred/aza |
| 12 | 19 | ENT/renal/lung | 150 | 73 | 20 | Pred/RTX |
| 13 | 3 | eye/joint | 11 | 79 | 40 | Pred/ aza |
| 14 | 12 | lung/ENT | 85 | 149 | 80 | Pred/RTX |
| 15 | 11 | renal/joints | 11 | 62 | 20 | Pred/CYC |
| 16 | 2 | joints | 34 | 75 | >640 | Pred |

ANCA, anti-neutrophil cytoplasmic antibody; Aza, azathioprine; BVAS, Birmingham Vasculitis Activity Score; CRP, C-reactive protein; Cyc, cyclophosphamide; ENT, ear, nose and throat; IIF, indirect immunofluorescence; MMF, mycophenolate mofetil; pred, prednisolone; RTX, rituximab
