# Supplementary figures and images for: Prospective monitoring of in vitro produced PR3-ANCA does not improve relapse prediction in granulomatosis with polyangiitis
Source: PLoS One. 2017 Aug 3;12(8):e0182549. doi: 10.1371/journal.pone.0182549 (PMC5542648; doi:10.1371/journal.pone.0182549)

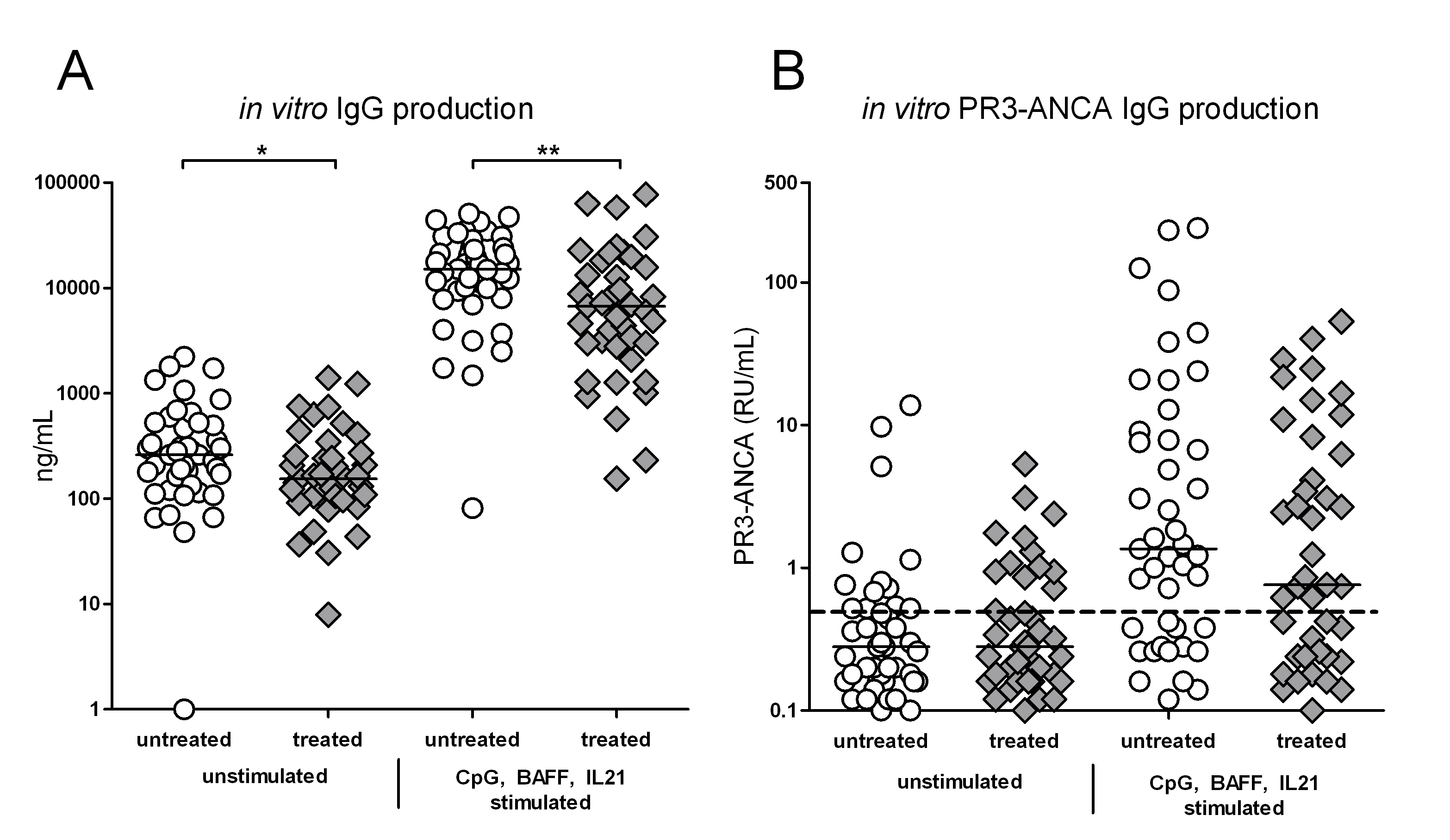

Supplement: S1 Fig — Graphs represent data of 84 GPA patients. Patients were divided based on whether they received immunosuppressive treatment. Patients classified as untreated are those that received no immunosuppression at time of sampling, all patients had received treatment in the past. All types of immunosuppressive treatment were combined in the treated group. A) Total IgG production was decreased in patients currently receiving treatment, while B) PR3-ANCA production was not significantly affected by current treatment. Horizontal lines represent median values. *p<0.05, **p<0.001. (TIF) [file pone.0182549.s002.tif]

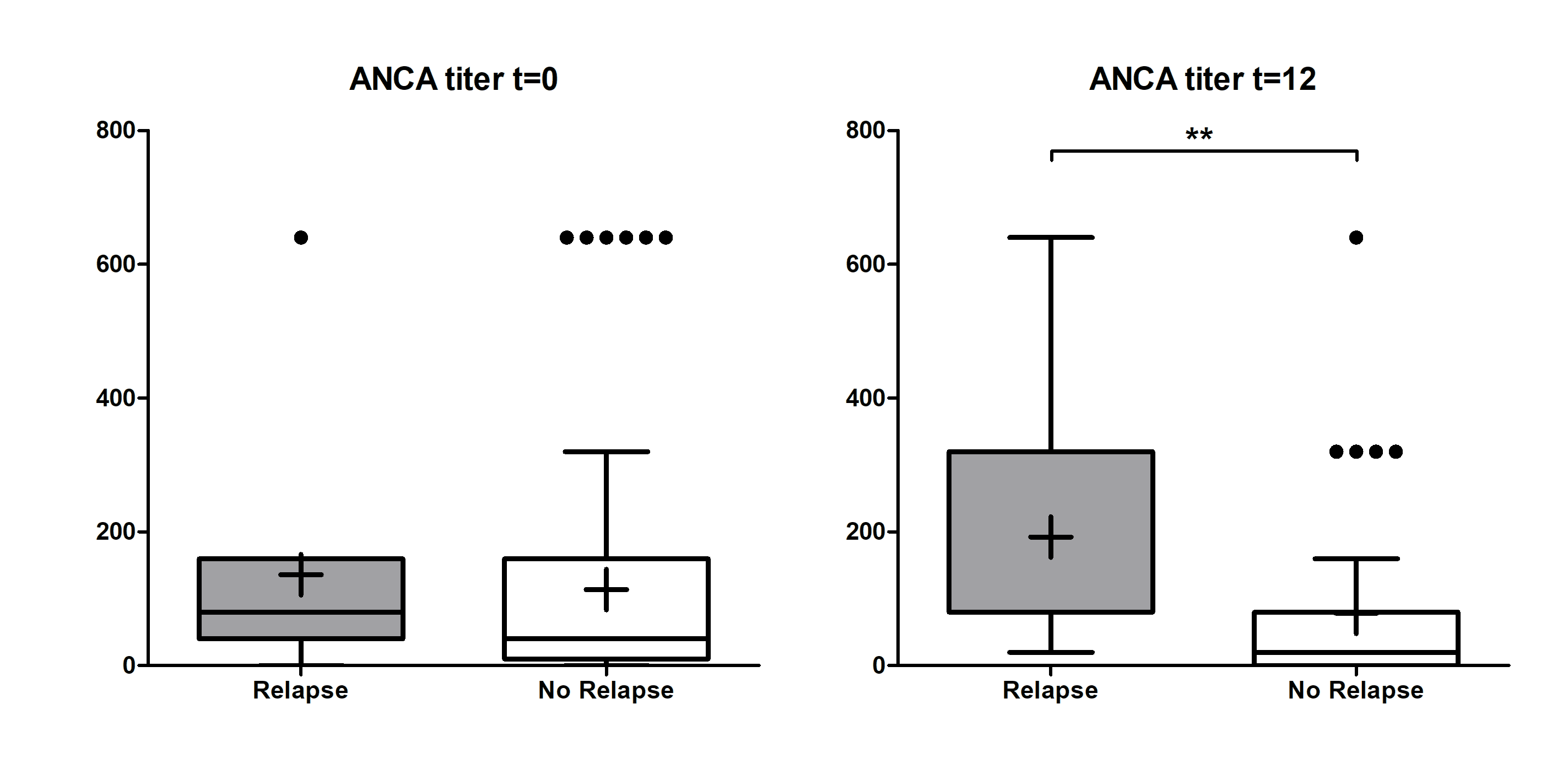

Supplement: S2 Fig — Graphs represent data of 84 GPA patients. Patients are divided based on whether they relapsed during the study period. In the left panel relapse and non-relapse patients are compared at time on inclusion, in the right panel after about 12 months. (TIF) [file pone.0182549.s003.tif]

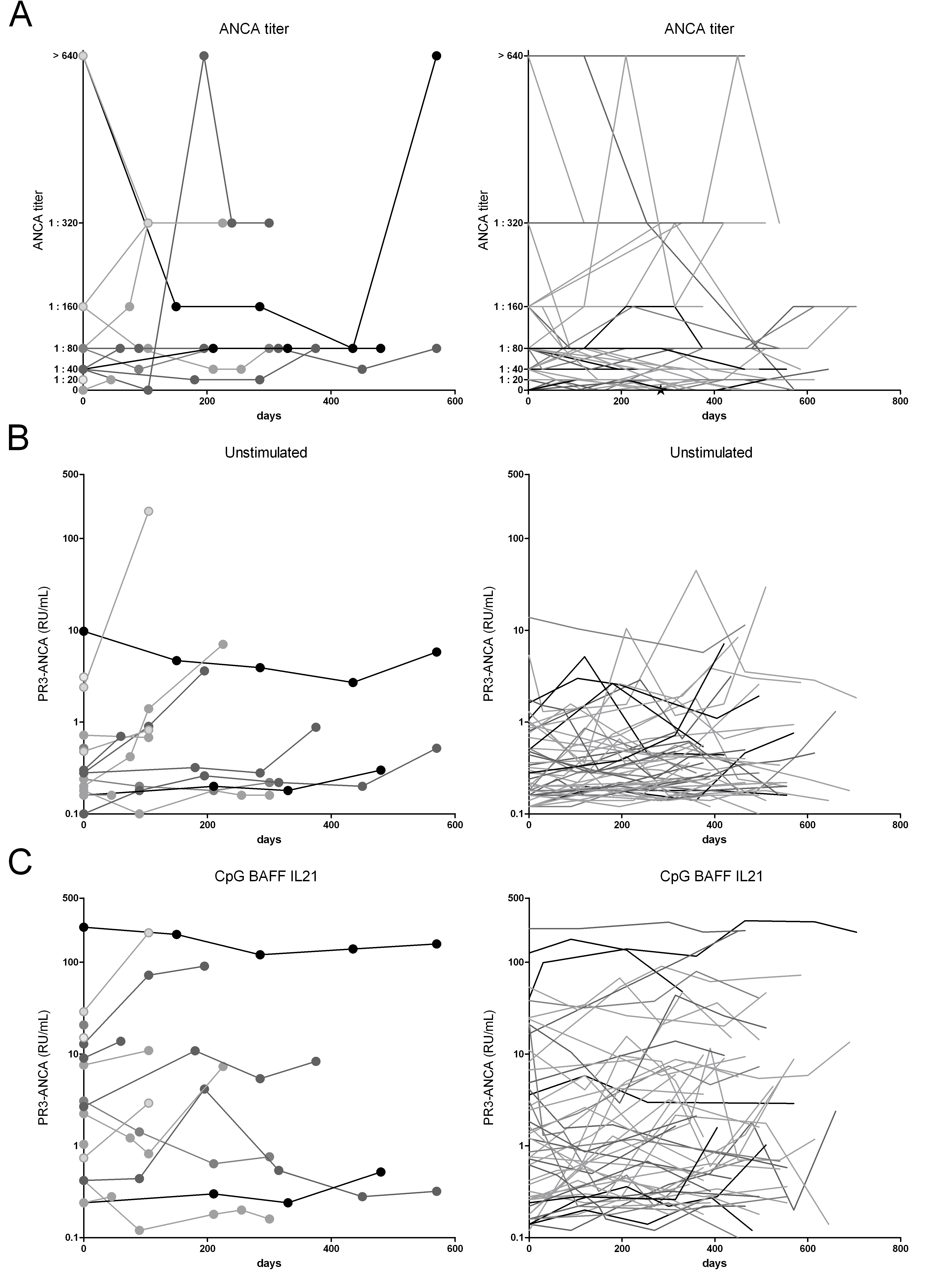

Supplement: S3 Fig — Results of all measured time points for A) ANCA titer, B) in vitro ANCA production in unstimulated culture samples and c) in vitro ANCA production in culture samples stimulated using CpG, BAFF and IL21 for individual patients. Graphs on the left represent 16 relapsing patients. Graphs on the right represent all non-relapsing patients with at least 3 samples during follow-up (n = 51). (TIF) [file pone.0182549.s004.tif]
